# Is endometriosis associated with poor sleep quality? A meta-analysis

**DOI:** 10.1177/17455057261446947

**Published:** 2026-04-29

**Authors:** Jillian Paul, Kim G. Harley

**Affiliations:** 1 Department of Epidemiology, School of Public Health, 40289 University of California at Berkeley, Berkeley, CA, USA

**Keywords:** endometriosis, sleep quality, chronic pain, inflammation, Women’s health, Women’s reproductive health

## Abstract

**Background:**

Endometriosis is a hormone-driven systemic inflammatory condition characterized by endometrial-like lesions which grow throughout the body affecting up to 15% of women worldwide with symptoms including chronic pain, infertility, and persistent fatigue. Many patients report a reduction in their quality of life, potentially driven in part by poor sleep quality. Despite this low sleep quality is not a commonly recognized symptom of endometriosis.

**Objective:**

We aimed to examine the association between endometriosis and poor sleep quality.

**Design:**

We conducted a systematic literature review and mixed-effects meta-analysis to evaluate the association between endometriosis and sleep quality.

**Data sources and methods:**

Six studies identified through PubMed and Embase met inclusion criteria and were assessed for quality. Between-group standardized mean differences (SMD) were pooled, and sensitivity analyses and meta-regressions were performed to evaluate the influence of between-study heterogeneity.

**Results:**

Pooling of all studies produced a standardized mean difference of 0.69 (95% CI: 0.28,1.09) with high heterogeneity (I^2^ = 94% p<0.01), which equates to a 2.44 (95%CI: 0.99; 3.85) point increase on the Pittsburg Sleep Quality Index (indicating poorer overall sleep quality) for endometriosis patients compared to healthy controls. This association persisted during sensitivity analyses.

**Conclusion:**

This observed association may be driven by a pain-sleep quality feedback loop or by the inflammation and hormonal imbalances of endometriosis. While there are several limitations to interpreting these results, including differences in control selections and adjustments for potential confounders between included studies, it is the first quantitative evaluation of the association between endometriosis and poor sleep quality across populations. Since sleep quality predicts chronic pain patterns, future research and patient treatment plans should focus on potential interventions to improve the sleep quality of endometriosis patients experience to reduce the societal and personal burdens of this disease.

## Plain language summary

Endometriosis is a chronic condition where tissue similar to the lining of the uterus grows outside of it, most often in the abdomen. It affects as many as one in seven women worldwide and can cause severe pain, fatigue, and infertility. Many people living with endometriosis also report problems with sleep, but poor sleep is not widely recognized as one of the condition’s symptoms or routinely studied by researchers. To better understand whether people with endometriosis experience worse sleep than those without the condition, we reviewed and combined the results of previous scientific studies that measured sleep quality in both groups. This process, called a systematic review and meta-analysis, helps summarize evidence from multiple studies to reach stronger conclusions. We identified six high-quality studies that met our inclusion criteria. When we analyzed them together, we found that people with endometriosis had significantly poorer sleep quality than those without it. On average, their scores on a standard sleep measure, the Pittsburgh Sleep Quality Index, were about 2.4 points higher, indicating worse overall sleep quality. This difference was consistent across several types of analyses, even though the studies varied in design and population. Our findings suggest that endometriosis may affect sleep through ongoing pain, inflammation, and hormonal changes, which can in turn worsen pain and fatigue, a cycle that may amplify the overall burden of the disease. Because good sleep is essential for physical and emotional well-being, these results highlight the need for more research on how endometriosis disrupts sleep and how improving sleep might reduce pain and improve quality of life for patients. Recognizing poor sleep as a common and meaningful symptom could lead to more holistic care for people living with endometriosis.

## Introduction

Endometriosis is a hormone-driven chronic systemic inflammatory condition, characterized by the growth of endometrial-like lesions throughout the body, that affects up to 15% of women worldwide.^1–3^ Symptomatology varies greatly among patients and is not correlated to the severity and number of endometriosis lesions.^4^ Common symptoms include severe abdominal pain, chronic musculoskeletal pain, persistent fatigue, gastrointestinal issues, and infertility.^5–7^ Additionally, endometriosis has been associated with a significant increase in depression and anxiety, with a 2023 study identifying a genetic link between endometriosis and these mental health conditions.^8–10^ Many endometriosis patients report severe reductions in quality of life due to both the mental and physical symptoms they experience.^11,12^ This reduction in quality of life associated with endometriosis has substantial negative impacts on both individuals and society as a whole. A 2018 study estimated that absenteeism and increased medical costs attributable to endometriosis across the United States lead to an annual financial impact of 69.4 billion dollars.^13^ In line with these results, a study of 6,925 endometriosis patients found that over 45% of patients reported that endometriosis pain impacted activities such as socializing and walking, and over 50% reported their symptoms impacted getting out of bed or working.^14^ Additionally, 42.6% of those patients reported endometriosis impacted their sleep quality. Despite these findings medical definitions and physicians rarely consider low sleep quality as a symptom of endometriosis.

Sleep quality is a well-established component of quality of life and studies show that poor sleep quality harms both mental and physical health.^15–17^ Overall sleep quality is generally evaluated on four aspects (sleep efficiency, sleep latency, sleep duration, and wake after sleep onset) and encompasses an individual’s satisfaction with these aspects as well.^18^ A disruption in any of these four areas can impact an individual’s daily life and is associated with an increased risk of short and long-term health conditions, including psychosocial issues and cardiovascular disease.^19^ Similarly, a 2024 study showed that after just two days of low sleep duration (4 hours in bed per night), individuals felt 4.44 years older compared to those who had 9 hours in bed per night.^20^ Poor sleep quality and related sleep disorders are present globally with prevalence varying significantly throughout populations; however, women consistently experience lower sleep quality than men, and low sleep quality is a primary complaint of over 65% of individuals with chronic pain conditions.^21–26^ The literature shows this association between chronic pain and low sleep quality is potentially bidirectional, but sleep quality tends to be a stronger predictor of chronic pain than the reverse.^27,28^

In 2024, Sumbodomet et al. conducted a qualitative review of the current literature on the potential association of endometriosis and sleep quality.^29^ The authors reported on nine papers that evaluated the difference in sleep quality between individuals with endometriosis and those without endometriosis. They reported evidence supporting an association between symptomatic endometriosis and poor sleep quality; however, no quantitative aggregation of sleep quality scores across studies was performed. Therefore, to draw more robust conclusions about the relationship and potential effect size across populations, and to better inform researchers and clinicians about sleep disturbance as an underrecognized symptom of endometriosis, we conducted a meta-analysis of studies evaluating quantitative differences in overall sleep quality between endometriosis patients and healthy individuals.

## Methods

### Search and selection

We performed a systematic literature search and review for papers related to sleep quality and endometriosis following PRISMA 2020 guidelines.^30^ The review and meta-analysis were registered with PROSPERO (CRD42024592272). Predefined objectives, eligibility criteria, the search strategy (PubMed Boolean logic and sleep-quality terms are provided in the manuscript), and data extraction procedures were determined before data extraction; a formal, standalone protocol or statistical analysis plan was not prepared. Generative AI was used to confirm the following Boolean search logic and related sleep quality terms were used to search PubMed^31^:

Endometriosis AND (“Sleep Disorders”[MeSH Terms] OR “Sleep Wake Disorders”[MeSH Terms] OR “Dyssomnias”[MeSH Terms] OR “Parasomnias”[MeSH Terms] OR “Insomnia”[MeSH Terms] OR “Sleep Apnea”[MeSH Terms] OR “Narcolepsy”[MeSH Terms] OR “Restless Legs Syndrome”[MeSH Terms] OR “Circadian Rhythm Sleep Disorders”[MeSH Terms] OR “Hypersomnia”[MeSH Terms] OR “Parasomnias”[MeSH Terms] OR “Sleep Bruxism”[MeSH Terms] OR “Sleep Paralysis”[MeSH Terms] OR “Sleep Disorders”[Text Word] OR “Sleep Disturbances”[Text Word] OR “Sleep Issues”[Text Word] OR “Sleep Problems”[Text Word] OR “Sleep Apnea Syndromes”[Text Word] OR “Obstructive Sleep Apnea”[Text Word] OR “Central Sleep Apnea”[Text Word] OR “Primary Insomnia”[Text Word] OR “Secondary Insomnia”[Text Word] OR “Narcolepsy with Cataplexy”[Text Word] OR “Narcolepsy without Cataplexy”[Text Word] OR “Willis-Ekbom Disease”[Text Word] OR “Delayed Sleep Phase Disorder”[Text Word] OR “Advanced Sleep Phase Disorder”[Text Word] OR “Non-24-Hour Sleep-Wake Disorder”[Text Word] OR “Shift Work Sleep Disorder”[Text Word] OR “Idiopathic Hypersomnia”[Text Word] OR “Sleepwalking”[Text Word] OR “Somnambulism”[Text Word] OR “Night Terrors”[Text Word] OR “Sleep Terrors”[Text Word] OR “REM Sleep Behavior Disorder”[Text Word] OR “Sleep Talking”[Text Word] OR “Somniloquy”[Text Word] OR “Sleep Quality”[Text Word] OR “Sleep Hygiene”[Text Word] OR “Sleep Deprivation”[Text Word] OR “Excessive Daytime Sleepiness”[Text Word] OR “Nightmares”[Text Word]).

Next, we conducted an Embase search based upon PICO search criteria with the population set as “endometriosis/exp,” intervention and comparison left blank, outcome set to “sleep quality/exp” with all synonyms included, and study design as “case control study/exp” to filter to studies which compared health controls with endometriosis patients. The last update to these searches was October 2025. First author (JP) reviewed titles and abstracts for inclusion, eliminating papers that were not peer-reviewed, not published in English, or unrelated to endometriosis and sleep quality. We also reviewed the citations of all papers marked for full-text review for further papers to include. During the full-text review, evaluated the remaining papers for inclusion based on the following criteria: non-ecologic study design, original data was reported, overall sleep quality was compared between endometriosis patients and healthy individuals on a quantitative scale, and the mean and standard deviation (SD) or median and interquartile range (IQR) of the sleep quality scores were reported for endometriosis patients and controls. A second author (KH) then independently review the inclusion and exclusion decisions as validation and no discrepancies were found. Authors who reported sleep quality on a quantitative scale but did not include the mean and standard deviation were contacted for further data (3 studies).

### Assessment of study quality

A traditional risk-of-bias assessment was not performed because these assessments have been shown to require highly subjective judgments and can be inconsistent and difficult to reproduce; instead authors jointly identified specific areas that could introduce bias into the aggregated results based on existing literature and the research.^32–35^ Author JP then assessed how each study’s potential risk of bias might influence the results of the meta-analysis in terms of direction (toward the null, no effect, away from the null, or potentially either) and magnitude (small, medium, large) and confirming these assertions with author KH through discussion. Disagreements were settled through use of existing literature and directed acrylic graphs as needed (Supplemental Table 1). Threats to potential selection bias were identified as whether controls were matched to cases, whether controls were screened for endometriosis, and whether there was a likelihood of Berkson’s bias due to use of hospital-based recruitment. Threats to potential information bias were determined to be greater when a scale other than the Pittsburgh Sleep Quality Index (PSQI), the predominant tool in most studies, was used, and if median/IQR was reported rather than mean/SD. The most important potential confounders were identified as age, body mass index, and hormonal therapy. Confounding was considered a potential source of bias if researchers either did not assess these variables or found a significant difference between cases and controls but did not account for it through adjustment, exclusion, or matching.

### Publication bias analysis

We planned to evaluate publication bias using funnel plot techniques, Begg’s rank test, and Egger’s regression test; however all methods were underpowered due to limited sample size of included papers.

### Data abstraction and preparation

For each study that met inclusion criteria, the following were extracted by author JP and reviewed by author KH: first author, year of publication, year(s) of data collection, the country where data was collected, the sample size for endometriosis patients and controls, method of endometriosis diagnosis, how controls were chosen and matched, which scale was used to measure sleep quality, covariates and potential confounders examined in the study or adjusted for in models, mean and SD or median and IQR of sleep quality scores for endometriosis patients and controls, whether power calculations were stated in the methodology, and any other quality-of-life measures examined (Supplemental Tables 2). The abstraction was conducted a second time, blinded to the first abstraction, for quality control purposes.

Data were standardized in preparation of the meta-analysis. To enable comparison between sleep scales, scales in which higher scores reflected better sleep quality were reversed by negating the means (mean = -mean), while standard deviations remained unchanged (SD = SD) so that higher scores consistently indicated poorer sleep quality.^36^ If a study reported median and interquartile range (IQR) for sleep scores, the median and IQR/1.35 were substituted for the mean and standard deviation.^36,37^

### Meta-analysis

We performed a mixed effect meta-analysis pooling between-group standardized mean differences (SMD) from all studies. We used Knapp-Hartung and Hedge’s g adjustments to account for anticipated high heterogeneity between studies.^38,39^ Results were expressed in a forest plot, and SMD was re-expressed for interpretability in terms of the most commonly used overall sleep quality scale, the PSQI. SMDs were multiplied by the average SD (3.534) in PSQI studies. We performed sensitivity analyses to examine how study variations may impact the results. First, we performed a leave-one-out analysis, excluding studies that did not use the PSQI scale to evaluate sleep quality. Next, we excluded studies that reported median and interquartile ranges instead of mean and SD.

To assess how study differences may impact results and heterogeneity, we performed a univariate meta-regression on the year data collection started and ended, the case and control sample sizes in each study, and the location of data collection. Statistical analyses were carried out using R Version 4.4.2, and the metaphor package.^40,41^

## Results

### Qualitative summary

**Figure 1.**
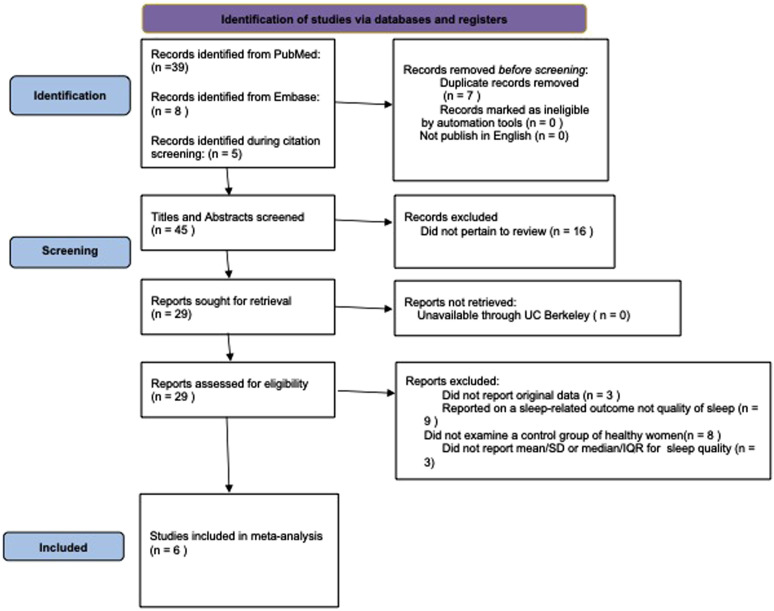
Literature search and review flowchart for selection of studies.

The literature search produced fifty-two potential papers (Figure 1), of which six met the inclusion criteria for the meta-analysis (Table 1).^42–47^ Three additional papers included an evaluation of endometriosis patients and healthy individuals but did not report mean/SD or median/IQR.^48–50^ The authors did not respond to requests for data and the papers were not included in the final 6. Research was performed in Brazil, Italy, Iran, and Spain. Five of the studies were conducted between 2016-2022; Nunes et al. 2015 was conducted earlier but did not report their collection years. All but one study used the PSQI, which represents better sleep quality with lower values, and the mean sleep scores ranged from 6.47 to 11.00 for endometriosis patients and 4.45 to 7.10 for healthy controls.^51^ Nunes et al. 2015 used the Post Sleep Inventory (PSI) scale, which represents better sleep quality with a higher number, and reported a mean sleep score of 5.68 in endometriosis patients and a mean of 6.04 in healthy controls.^52^ Sample size varied considerably between studies. Álvarez-Salvago et al., 2020 reported on the smallest study of 25 cases and 25 controls, while Iannuzzo et al., 2024 reported on the largest sample of 430 cases and 417 controls. All six studies reported statistically significantly poorer average sleep quality among endometriosis patients compared to healthy controls.

**Table 1.** Studies included in the meta-analysis of endometriosis and sleep quality and their characteristics.

Reference	Location	Study years	Sample size		Sleep quality scale	Sleep quality scores (mean, SD)	
			Endo	Control		Endo	Control
Álvarez-Salvago, 2020^42^	Spain	2016-2017	25	25	PSQI	8.32 (3.73)	6 (3.19)
Chaichian, 2024^43^	Iran	2019-2022	46	202	PSQI	10.60 (3.70)	7.1 (2.7)
Facchin, 2021^44^	Italy	2019-2020	123	123	PSQI	6.68 (3.59)	5.45 (3.03)
Iannuzzo, 2024**,^45^	Italy	2021	430	417	PSQI	11.00 (6.67)	5.00 (2.20)
Nunes, 2015^46^	Brazil	NR	257	253	PSI*	5.68 (1.55)	6.04 (1.62)
Youseflu, 2020^47^	Iran	2016-2017	78	78	PSQI	6.47 (3.34)	4.45 (3.26)

*Score on original scale PSI was reverse coded for analysis.

**Reported median and interquartile range.

Endo=Endometriosis, NR=Not Reported, PSQI=Pittsburgh Sleep Quality Index, PSI=Post-Sleep Inventory.

Potential threats to bias varied across the included studies (Table 2). The most common area for potential bias was selection bias, with four of the six studies having one or more areas of potential selection bias identified. Two studies (Iannuzzo et al., 2024 and Nunes et al., 2015) were identified as having potential information bias threats. Four studies had potential confounding bias, primarily due to a lack of assessing or controlling for the use of hormonal therapies in endometriosis patients. One study (Facchin et al., 2021) met all requirements to avoid potential bias. The potential sources of bias identified in Nunes et al., 2015 were assessed as having a moderate impact on the results, potentially shifting them in either direction from the null depending on the strength of the biases. Chaichian et al., 2024 had the largest risk of bias toward the null due to high chance of selection bias and lack of screening for endometriosis in controls. Two studies (Iannuzo et al., 2024 and Youseflu et al., 2020) were scored to have a moderate risk of impact toward the null due to selection and information bias, and Berkson’s bias respectively. Alvarez-Salvago et al., 2020 was evaluated to have a small risk of bias toward the null due to potential confounding through HRT.

**Table 2.** Assessment of threats to validity across studies included in meta-analysis.

​	Potential selection bias		Potential information bias			Potential confounding bias*			Impact on study	
Reference	Matching of controls	Berkson’s bias	Controls screened for endo	PSQI sleep scale used	Reported mean and SD	Age assessed/controlled for	BMI assessed/controlled for	Use of HT assessed/controlled for	Magnitude	Direction
Álvarez-Salvago, 2020^42^	✓	✓	✓	✓	✓	✓	✓	**X**	small	toward null
Chaichian, 2024^43^	**X**	**X**	**X**	✓	✓	✓	✓	**X**	large	toward null
Facchin, 2021^44^	✓	✓	✓	✓	✓	✓	✓	✓	none	none
Iannuzzo, 2024^45^	**X**	✓	**X**	✓	**X**	✓	✓	✓	moderate	toward null
Nunes, 2015^46^	**X**	✓	**X**	**X**	✓	**X**	**X**	✓	moderate	Potentially either
Youseflu, 2020^47^	✓	**X**	✓	✓	✓	✓	✓	**X**	moderate	toward null

✓= avoided potential bias.

**X** = did not avoid potential bias.

Endo=endometriosis, PSQI= Pittsburgh Sleep Quality Index, SD= standard deviation, BMI= Body Mass Index, HT = hormonal therapy.

*Confounding was considered as at risk for potential bias if researchers did not compare the distributions of the variables between cases and controls, or they did compare the distributions, found a significant difference between cases and controls, but the variable was not adjusted for, excluded on, or matched upon.

### Meta-analysis

**Figure 2.**
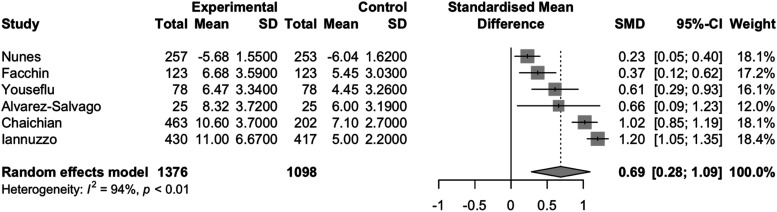
Forest plot of the standardized mean difference meta-analysis of studies comparing sleep quality of endometriosis patients to healthy controls.

**Figure 3.**
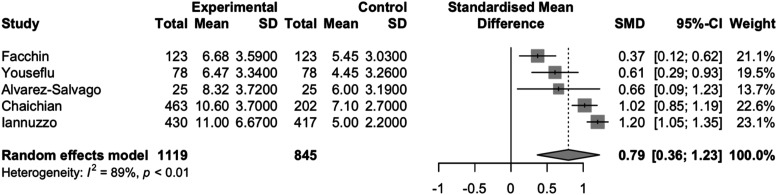
Forest Plot of the standardized mean difference meta-analysis of studies comparing sleep quality of endometriosis patients to healthy controls, excluding study that did not use the pittsburgh sleep quality index.

**Figure 4.**
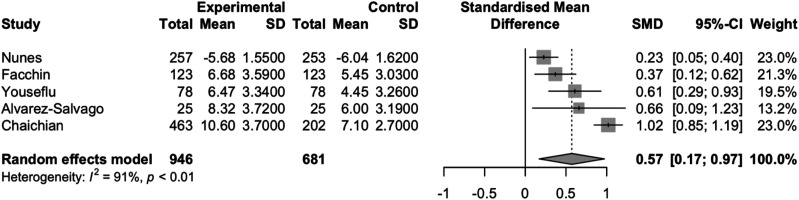
Forest plot of the standardized mean difference meta-analysis of studies comparing sleep quality of endometriosis patients to healthy controls, excluding study that reported median and interquartile range.

The pooling of all six studies produced a standardized mean difference of 0.69 (95% CI: 0.28,1.09) with high heterogeneity (I^2^ = 94% p<0.01), which equates to a 2.44 (95%CI: 0.99, 3.85) point increase on the PSQI scale for endometriosis patients compared to healthy controls (Figure 2). When the single study that did not use the PSQI scale was removed from the meta-analysis, the pooled SMD was 0.79 (95% CI:0.36,1.23), which represents a 2.79 (95% CI: 1.27, 4.35) point increase on the PSQI scale for endometriosis patients compared to healthy controls with high heterogeneity between studies (I^2^: 89% p<0.01) (Figure 3). When the study that reported median and IQR was removed, the pooled SMD was 0.57 (95% CI: 0.17, 0.97) with high heterogeneity (I^2^ = 91%, p<0.01), representing a 2.01 (95% CI: 0.60,3.43) point increase on the PSQI scale for endometriosis patients compared to healthy controls (Figure 4).

Heterogeneity between studies remained high in univariate meta-regression analyses (I^2^ ranging from 79.4%-90.59%) (Table 3). Meta-analysis results indicated much of the differences in true effect sizes were explained by the location of data collection (53.04%), the sample size of endometriosis patients (35.96%), and the year of study (year started: 17.51%, year ended:14.32%).

**Table 3.** Results of meta-regression examining how study characteristics impacted heterogeneity (I^2^) of studies included in meta-analysis.

Study characteristic	Difference in effect size (R ^2^ )	Residual heterogeneity (I ^2^ )	Reduction in heterogeneity (94% - I ^2^ )
Location of Data Collection	53.04%	79.41%	14.59%
Year Study Began	17.51%	85.8%	8.92%
Year Study ended	14.32%	87.56%	6.44%
Endometriosis patient sample size	35.96%	88.16%	5.84%
Control sample size	8.13%	90.59%	3.41%

R^2^= Measure of difference in effect size between studies included in meta-analysis, I^2^= Measure of heterogeneity between studies included in meta-analysis.

### Publication bias

Due to a small number of studies that met inclusion criteria, publication bias was not evaluated as funnel plots and regression-based methods were all underpowered.

## Discussion

This, to the best of the authors’ knowledge, represents the first quantitative evaluation of the association between endometriosis and poor sleep quality across populations through a meta-analysis. The 2.44 point increase in the average PSQI score, which ranges from 0-21 with good sleep quality scoring between 0 and 5 of endometriosis patients compared to individuals without the condition indicates a potential association between endometriosis and reduced sleep quality.

This measure of association further supports and elucidates the association examined by the current literature. Sumbodo et al. 2024 found evidence of an association between endometriosis and sleep disturbances in their narrative synthesis. Meanwhile, two studies have found that surgical interventions significantly improved the sleep quality of endometriosis patients compared to before surgery.^53,54^ In several survey-based studies, endometriosis patients have included low sleep quality as a personal burden experienced in connection to their symptoms.^14,55,56^ Other studies have examined how endometriosis symptoms, including poor sleep quality, have impacted daily activities such as the ability to work.^57,58^ This body of research indicates that endometriosis is likely associated with poor sleep quality, highlighting and documenting a burden of disease that should be further explored as a clinical symptom and target in treatment plans.

The mechanisms by which this association may occur are unclear. However, research into other chronic pain conditions, such as fibromyalgia, arthritis, and back pain, suggests that chronic pain activates the hypothalamic-pituitary-adrenal (HPA) axis, increases sleep disturbances and inflammation in the body, and creates a feedback loop between pain and lowered sleep quality.^59,60^ It is reasonable to assume a similar effect occurs in endometriosis patients, potentially further amplified by the inflammatory nature of endometriosis.

Furthermore, research has begun to show that endometriosis’s immune and hormonal dysregulations may be directly interrupting sleep cycles. Pro-inflammatory cytokines, which play an important role in the progression of endometriosis, have been identified as a potential link between chronic pain and sleep disturbances.^61^ Fluctuations in female-reproductive hormones that influence sleep and circadian rhythms may be negatively amplified by the estrogen-dependent imbalances of endometriosis.^1,3,62^ Anecdotally, endometriosis patients report higher sleep disturbances during times of their menstrual cycle when progesterone and estrogen are rapidly changing. Studies interested in this potential mechanism have begun to examine the impact of melatonin, a hormone related to both sleep and the female reproductive system, has on endometriosis patients, with a phase II double-blind clinical trial finding both an improvement in sleep quality and a reduction in pain in patients treated with melatonin.^63–65^

Additionally, comorbidities, particularly depression and anxiety, which are both highly prevalent in people with endometriosis, may modify the relationship between endometriosis and overall sleep quality. These conditions have been shown to independently worsen sleep and could confound or mediate the associations observed. Finally, a bidirectional relationship is plausible: poor sleep can amplify pain sensitivity, inflammatory signaling, and mood symptoms, which in turn may worsen endometriosis-related symptoms and further degrade sleep. This ‘feedback loop’ has been observed in other chronic pain conditions and highlights the need for longitudinal studies and mediation analyses to disentangle confounding, mediation, and directional causality.

## Limitations

Several limitations to this study should be considered when evaluating the results. The potential for selection bias was high among the included studies. Most studies were of a cross-sectional nature limiting the ability to interpret causally. Many studies did not screen for endometriosis in controls, potentially including undiagnosed individuals and biasing results toward the null. Adjustments for confounding variables varied greatly between included studies. In particular, half of the included studies did not assess or control for the use of hormonal therapies despite evidence that these treatments improve sleep quality by regulating hormones in menopausal women.^59,60,66,67^ Given that hormonal therapy is a common treatment for endometriosis symptoms and is likely to be more prevalent among cases than controls, its omission may bias the results toward the null. The small sample size of included studies and high heterogeneity between studies make the effect estimate less interpretable than initially anticipated. Additionally, despite meta-regressions which elucidating potential factors leading to this high heterogeneity small sample size of included studies limited the ability to further subgroup by these areas. There is also a high potential for publication bias in the literature, which we were unable to evaluate based on sample size and power.

Future research on endometriosis and overall sleep quality should aim to strengthen the ability to evaluate the causal relationship between endometriosis and poor sleep quality. Particularly longitudinal studies that properly adjust for variations in individual symptom burdens, potential confounders, including the use of hormonal therapy, comorbidities know to impact overall sleep quality including depression and anxiety, and the ability to assess the potential for a bi-directional relationship between overall sleep quality and endometriosis. In addition, controls should be properly screened for endometriosis or other chronic pain comorbidities to reduce non-differential misclassification.

Despite these limitations, the study has numerous strengths that should be noted. The systematic and comprehensive nature of the review and analysis allows for examining associations across multiple populations and increases the generalizability of findings. The sensitivity analysis and meta-regressions indicate the effect estimate is potentially robust despite limitations related to sample size and heterogeneity. The included studies enrolled endometriosis patients who were diagnosed months or years before their sleep evaluations, minimizing concerns that poor sleep quality could exacerbate endometriosis symptoms through reverse causality. Lastly, this study has quantitatively evaluated an association that has only been summarized qualitatively and supports patterns emerging in the literature.

## Conclusion

This study highlights the potential for significant and clinically meaningful associations between endometriosis and reductions in sleep quality, which should be further investigated in ongoing high quality longitudinal research. Since sleep quality predicts chronic pain patterns, a better understanding of overall sleep quality in endometriosis patients may lead to improved patient care and treatments, helping to reduce pain, improve individuals’ quality of life, and decrease the overall burden of endometriosis on society.

## Supplemental material

Supplemental material - Is endometriosis associated with poor sleep quality? A meta-analysis Supplemental material for Is endometriosis associated with poor sleep quality? A meta-analysis by Jillian Paul and Kim G. Harley in Women’s Health.

## Data Availability

Data are available in previously published papers the project was abstracted from.*

## References

[1] Chauhan S More A Chauhan V , et al. Endometriosis: A Review of Clinical Diagnosis, Treatment, and Pathogenesis. Cureus 2022; 14: e28864. 10.7759/cureus.28864 36225394 PMC9537113

[2] Cano-Herrera G Salmun Nehmad S Ruiz de Chávez Gascón J , et al. Endometriosis: A Comprehensive Analysis of the Pathophysiology, Treatment, and Nutritional Aspects, and Its Repercussions on the Quality of Life of Patients. Biomedicines 2024; 12: 1476. 10.3390/biomedicines12071476 39062050 PMC11274817

[3] Wang Y Nicholes K Shih I-M . The Origin and Pathogenesis of Endometriosis. Annu Rev Pathol Mech Dis 2020; 15: 71–95. 10.1146/annurev-pathmechdis-012419-032654 PMC7980953 31479615

[4] Warzecha D Szymusik I Wielgos M , et al. The Impact of Endometriosis on the Quality of Life and the Incidence of Depression—A Cohort Study. Int J Environ Res Public Health 2020; 17: 3641. 10.3390/ijerph17103641 32455821 PMC7277332

[5] Nagy H Carlson K Khan MA . Dysmenorrhea. StatPearls. StatPearls Publishing, 2024. https://www.ncbi.nlm.nih.gov/books/NBK560834/ (accessed 16 September 2024). 32809669

[6] Tsamantioti ES Mahdy H . Endometriosis. StatPearls. StatPearls Publishing, 2024. https://www.ncbi.nlm.nih.gov/books/NBK567777/ (accessed 16 September 2024). 33620854

[7] Saunders PTK Horne AW . Endometriosis: Etiology, pathobiology, and therapeutic prospects. Cell 2021; 184: 2807–2824. 10.1016/j.cell.2021.04.041 34048704

[8] Missmer SA Tu F Soliman AM , et al. Impact of endometriosis on women’s life decisions and goal attainment: a cross-sectional survey of members of an online patient community. BMJ Open 2022; 12: e052765. 10.1136/bmjopen-2021-052765 PMC9047767 35477879

[9] Koller D Pathak GA Wendt FR , et al. Epidemiologic and Genetic Associations of Endometriosis With Depression, Anxiety, and Eating Disorders. JAMA Netw Open 2023; 6: e2251214. 10.1001/jamanetworkopen.2022.51214 36652249 PMC9856929

[10] Gambadauro P Carli V Hadlaczky G . Depressive symptoms among women with endometriosis: a systematic review and meta-analysis. Am J Obstet Gynecol 2019; 220: 230–241. 10.1016/j.ajog.2018.11.123 30419199

[11] Maulenkul T Kuandyk A Makhadiyeva D , et al. Understanding the impact of endometriosis on women’s life: an integrative review of systematic reviews. BMC Womens Health 2024; 24: 524. 10.1186/s12905-024-03369-5 39300399 PMC11411992

[12] Young K Fisher J Kirkman M . Women’s experiences of endometriosis: a systematic review and synthesis of qualitative research. J Fam Plann Reprod Health Care 2015; 41: 225–234. 10.1136/jfprhc-2013-100853 25183531

[13] Soliman AM Yang H Du EX , et al. The direct and indirect costs associated with endometriosis: a systematic literature review. Hum Reprod 2016; 31: 712–722. 10.1093/humrep/dev335 26851604

[14] Ensari I Pichon A Lipsky-Gorman S , et al. Augmenting the Clinical Data Sources for Enigmatic Diseases: A Cross-Sectional Study of Self-Tracking Data and Clinical Documentation in Endometriosis. Appl Clin Inform 2020; 11: 769–784. 10.1055/s-0040-1718755 33207385 PMC7673957

[15] Krystal AD Edinger JD . Measuring sleep quality. Sleep Med 2008; 9: S10–S17. 10.1016/S1389-9457(08)70011-X 18929313

[16] Scott AJ Webb TL James MM-S , et al. Improving sleep quality leads to better mental health: A meta-analysis of randomised controlled trials. Sleep Med Rev 2021; 60: 101556. 10.1016/j.smrv.2021.101556 34607184 PMC8651630

[17] Gao C Guo J Gong T-T , et al. Sleep Duration/Quality With Health Outcomes: An Umbrella Review of Meta-Analyses of Prospective Studies. Front Med 8: 813943. doi:10.3389/fmed.2021.813943, Epub ahead of print 20 January 2022. PMC8811149 35127769

[18] Nelson KL Davis JE Corbett CF . Sleep quality: An evolutionary concept analysis. Nurs Forum (Auckl) 2022; 57: 144–151. 10.1111/nuf.12659 34610163

[19] Medic G Wille M Hemels ME . Short- and long-term health consequences of sleep disruption. Nat Sci Sleep 2017; 9: 151–161. 10.2147/NSS.S134864 28579842 PMC5449130

[20] Balter LJT Axelsson J . Sleep and subjective age: protect your sleep if you want to feel young. Proc R Soc B Biol Sci 2024; 291: 20240171. 10.1098/rspb.2024.0171 PMC10965331 38531399

[21] Theorell-Haglöw J Miller CB Bartlett DJ , et al. Gender differences in obstructive sleep apnoea, insomnia and restless legs syndrome in adults – What do we know? A clinical update. Sleep Med Rev 2018; 38: 28–38. 10.1016/j.smrv.2017.03.003 28495359

[22] Kohanmoo A Kazemi A Zare M , et al. Gender-specific link between sleep quality and body composition components: a cross-sectional study on the elderly. Sci Rep 2024; 14: 8113. 10.1038/s41598-024-58801-5 38582755 PMC10998859

[23] Alosta MR Oweidat I Alsadi M , et al. Predictors and disturbances of sleep quality between men and women: results from a cross-sectional study in Jordan. BMC Psychiatry 2024; 24: 200. 10.1186/s12888-024-05662-x 38475779 PMC10936022

[24] Fatima Y Doi SA Najman JM , et al. Exploring Gender Difference in Sleep Quality of Young Adults: Findings from a Large Population Study. Clin Med Res 2016; 14: 138–144. 10.3121/cmr.2016.1338 28188139 PMC5302457

[25] Duo L Yu X Hu R , et al. Sleep disorders in chronic pain and its neurochemical mechanisms: a narrative review. Front Psychiatry 2023; 14: 1157790. 10.3389/fpsyt.2023.1157790 37324825 PMC10267346

[26] Smith MT Haythornthwaite JA . How do sleep disturbance and chronic pain inter-relate? Insights from the longitudinal and cognitive-behavioral clinical trials literature. Sleep Med Rev 2004; 8: 119–132. 10.1016/S1087-0792(03)00044-3 15033151

[27] Runge N Ahmed I Saueressig T , et al. The bidirectional relationship between sleep problems and chronic musculoskeletal pain: a systematic review with meta-analysis. Pain 2024; 165: 2455–2467. 10.1097/j.pain.0000000000003279 38809241

[28] Koffel E Kroenke K Bair MJ , et al. The bidirectional relationship between sleep complaints and pain: Analysis of data from a randomized trial. Health Psychol 2016; 35: 41–49. 10.1037/hea0000245 26076002 PMC4900176

[29] Sumbodo CD Tyson K Mooney S , et al. The relationship between sleep disturbances and endometriosis: A systematic review. Eur J Obstet Gynecol Reprod Biol 2024; 293: 1–8. 10.1016/j.ejogrb.2023.12.010 38091847

[30] Page MJ McKenzie JE Bossuyt PM , et al. The PRISMA 2020 statement: An updated guideline for reporting systematic reviews. PLoS Med 2021; 18: e1003583. 10.1371/journal.pmed.1003583 33780438 PMC8007028

[31] ChatGPT . ChatGPT. https://chatgpt.com/ (accessed 27 March 2026).

[32] Arroyave WD Mehta SS Guha N , et al. Challenges and recommendations on the conduct of systematic reviews of observational epidemiologic studies in environmental and occupational health. J Expo Sci Environ Epidemiol 2021; 31: 21–30. 10.1038/s41370-020-0228-0 32415298 PMC7666644

[33] Savitz DA Wellenius GA Trikalinos TA . The Problem With Mechanistic Risk of Bias Assessments in Evidence Synthesis of Observational Studies and a Practical Alternative: Assessing the Impact of Specific Sources of Potential Bias. Am J Epidemiol 2019; 188: 1581–1585. 10.1093/aje/kwz131 31145434

[34] Steenland K Schubauer-Berigan MK Vermeulen R , et al. Risk of Bias Assessments and Evidence Syntheses for Observational Epidemiologic Studies of Environmental and Occupational Exposures: Strengths and Limitations. Environ Health Perspect 2020; 128: 95002. 10.1289/EHP6980 32924579 PMC7489341

[35] Greenland S O’Rourke K . On the bias produced by quality scores in meta-analysis, and a hierarchical view of proposed solutions. Biostat Oxf Engl 2001; 2: 463–471. 10.1093/biostatistics/2.4.463 12933636

[36] Chapter 6 . Choosing effect measures and computing estimates of effect. https://training.cochrane.org/handbook/current/chapter-06 (accessed 30 October 2024).

[37] Wan X Wang W Liu J , et al. Estimating the sample mean and standard deviation from the sample size, median, range and/or interquartile range. BMC Med Res Methodol 2014; 14: 135. 10.1186/1471-2288-14-135 25524443 PMC4383202

[38] Taylor JM Alanazi S . Cohen’s and Hedges’ g. J Nurs Educ 2023; 62: 316–317. 10.3928/01484834-20230415-02 37146047

[39] Harrer M Cuijpers P Furukawa TA , et al. Chapter 4 Pooling Effect Sizes Doing Meta-Analysis in R. https://bookdown.org/MathiasHarrer/Doing_Meta_Analysis_in_R/pooling-es.html (accessed 3 December 2024).

[40] R Core Team . R: A language and environment for statistical computing. R Foundation for Statistical Computing, 2023.

[41] Viechtbauer W . metafor: Meta-Analysis Package for R. https://cran.r-project.org/web/packages/metafor/index.html (2025, accessed 18 February 2025).

[42] Álvarez-Salvago F Lara-Ramos A Cantarero-Villanueva I , et al. Chronic Fatigue, Physical Impairments and Quality of Life in Women with Endometriosis: A Case-Control Study. Int J Environ Res Public Health 2020; 17: 3610. 10.3390/ijerph17103610 32455618 PMC7277433

[43] Chaichian S Mehdizadehkashi A Haghgoo A , et al. Sleep disorders in patients with endometriosis; a cross-sectional study. BMC Womens Health 2024; 24: 1–7. 10.1186/s12905-024-03185-x 38877485 PMC11177365

[44] Facchin F Buggio L Roncella E , et al. Sleep disturbances, fatigue and psychological health in women with endometriosis: a matched pair case–control study. Reprod Biomed Online 2021; 43: 1027–1034. 10.1016/j.rbmo.2021.08.011 34756643

[45] Iannuzzo F Garzon S Lazzari C , et al. Sleep disorders and hyperarousal among patients with endometriosis: A case-control survey study. Eur J Obstet Gynecol Reprod Biol 2024; 300: 287–295. 10.1016/j.ejogrb.2024.07.031 39053089

[46] Nunes Fr. Ferreira Jm. Bahamondes L . Pain threshold and sleep quality in women with endometriosis. Eur J Pain 2015; 19: 15–20. 10.1002/ejp.514 24733758

[47] Youseflu S Jahanian Sadatmahalleh S Roshanzadeh G , et al. Effects of endometriosis on sleep quality of women: does life style factor make a difference? BMC Womens Health 2020; 20: 1–7. 10.1186/s12905-020-01036-z 32778090 PMC7418319

[48] Chmaj-Wierzchowska K Rzymski P Wojciechowska M , et al. Health-related quality of life (Nottingham Health Profile) in patients with endometriomas: correlation with clinical variables and self-reported limitations. Arch Med Sci AMS 2019; 16: 584–591. 10.5114/aoms.2019.82744 32399106 PMC7212235

[49] Davie S Hamilton Y Webb L , et al. Sleep quality and endometriosis: A group comparison study. J Endometr Pelvic Pain Disord 2020; 12: 94–100. 10.1177/2284026520909979

[50] Leone Roberti Maggiore U Bizzarri N Scala C , et al. Symptomatic endometriosis of the posterior cul-de-sac is associated with impaired sleep quality, excessive daytime sleepiness and insomnia: a case–control study. Eur J Obstet Gynecol Reprod Biol 2017; 209: 39–43. 10.1016/j.ejogrb.2015.11.026 26700500

[51] Buysse DJ Reynolds CF Monk TH , et al. The Pittsburgh sleep quality index: A new instrument for psychiatric practice and research. Psychiatry Res 1989; 28: 193–213. 10.1016/0165-1781(89)90047-4 2748771

[52] Webb WB Bonnet M Blume G . A post-sleep inventory. Percept Mot Skills 1976; 43: 987–993. 10.2466/pms.1976.43.3.987

[53] Goksu M Kadirogullari P Seckin KD . Evaluation of depression and sleep disorders in the preoperative and postoperative period in stage 4 endometriosis patients. Eur J Obstet Gynecol Reprod Biol 2021; 264: 254–258. 10.1016/j.ejogrb.2021.07.037 34333367

[54] Halici BNA Aktoz F Kabakci M , et al. Analysis of preoperative and postoperative quality of life, sexual function, and sleep in patients with endometriosis: a prospective cohort study. Arch Gynecol Obstet 2023; 307: 113–120. 10.1007/s00404-022-06562-9 35451649

[55] de Souza RJ Brollo LCS Carrerette FB , et al. Challenges in measuring sleep quality among women with endometriosis: A comparison of two questionnaires. Sleep Med 2024; 114: 250–254. 10.1016/j.sleep.2024.01.008 38244462

[56] Davari-Tanha F Askari F Akrami M , et al. Sleep Quality in Women with Endometriosis. Acad J Surg 2014; 1: 57–59.

[57] Mundo-López A Ocón-Hernández O Lozano-Lozano M , et al. Impact of symptom burden on work performance status in Spanish women diagnosed with endometriosis. Eur J Obstet Gynecol Reprod Biol 2021; 261: 92–97. 10.1016/j.ejogrb.2021.04.008 33906026

[58] Fourquet J Gao X Zavala D , et al. Patients’ report on how endometriosis affects health, work, and daily life. Fertil Steril 2010; 93: 2424–2428. 10.1016/j.fertnstert.2009.09.017 19926084 PMC2860000

[59] As-Sanie S Shafrir AL Halvorson L , et al. The Burden of Pelvic Pain Associated With Endometriosis Among Women in Selected European Countries and the United States: A Restricted Systematic Review. J Minim Invasive Gynecol 2024; 31: 653–666.e5. 10.1016/j.jmig.2024.05.002 38729420

[60] Seiger AN Penzel T Fietze I . Chronic pain management and sleep disorders. Cell Rep Med 2024; 5: 101761. 10.1016/j.xcrm.2024.101761 39413729 PMC11513819

[61] Haack M Simpson N Sethna N , et al. Sleep deficiency and chronic pain: potential underlying mechanisms and clinical implications. Neuropsychopharmacol Off Publ Am Coll Neuropsychopharmacol 2020; 45: 205–216. 10.1038/s41386-019-0439-z PMC6879497 31207606

[62] Nowakowski S Meers J Heimbach E . Sleep and Women’s Health. Sleep Med Res 2013; 4: 1–22. 10.17241/smr.2013.4.1.1 25688329 PMC4327930

[63] Schwertner A Conceição dos Santos CC Costa GD , et al. Efficacy of melatonin in the treatment of endometriosis: A phase II, randomized, double-blind, placebo-controlled trial. PAIN® 2013; 154: 874–881. 10.1016/j.pain.2013.02.025 23602498

[64] Hoseinalizadeh H Cahichian S . Evaluation of melatonin effect on pelvic pain in women with endometriosis referred to affiliated hospitals to Tehran Medical Sciences of Islamic Azad University. Med Sci J Islam Azad Univesity - Tehran Med Branch 2018; 28: 277–282. 10.29252/iau.28.4.277

[65] Li Q Zheng T Chen J , et al. Exploring melatonin’s multifaceted role in female reproductive health: From follicular development to lactation and its therapeutic potential in obstetric syndromes. J Adv Res 2024; 70: 223. 10.1016/j.jare.2024.04.025, Epub ahead of print 30. 38692429 PMC11976432

[66] Sarti CD Chiantera A Graziottin A , et al. Hormone therapy and sleep quality in women around menopause. Menopause 2005; 12: 545–551. 10.1097/01.gme.0000172270.70690.5e 16145308

[67] Cintron D Lahr BD Bailey KR , et al. Effects of oral versus transdermal menopausal hormone treatments on self-reported sleep domains and their association with vasomotor symptoms in recently menopausal women enrolled in the Kronos Early Estrogen Prevention Study (KEEPS). Menopause 2018; 25: 145–153. 10.1097/GME.0000000000000971 28832429 PMC5771895

