# Supplementary material for: Is endometriosis associated with poor sleep quality? A meta-analysis
Source: Womens Health (Lond). 2026 Apr 29;22:17455057261446947. doi: 10.1177/17455057261446947 (PMC13150089; doi:10.1177/17455057261446947)
Supplement: Supplemental material - Is endometriosis associated with poor sleep quality? A meta-analysis [file sj-pdf-1-whe-10.1177_17455057261446947.pdf]

Supplemental Table 1: Directional impact of factors identified as potential threats to validity

Matching of controls	Controls screened for endo	Berkson's bias	PSQI sleep scale used	Reported mean and SD	Age assessed/ controlled for	BMI assessed/ controlled for	Use of HT assessed/ controlled for
Either direction depending on matching - less weighting if the confounders were well handled	Toward the Null Potential misclassification with endo patients showing up in controls	Toward the Null Was not considered as a threat if controls were collected from standard OBGYN visit	Could vary depending on the other scale used PSQI used as reference because it was the most prevalent and well validated	Reduces precision of the effect estimate	Toward the Null More likely to be diagnosed with endo at an older age and age reduces sleep quality	Away from Null More likely to be diagnosed with lower BMI and higher sleep quality with lower BMI	Toward the Null More likely to be used in endometriosis patients and masks symptoms

Endo= Endometriosis, SD = Standard Deviation, BMI= Body Mass Index, HT= Hormonal Therapy, OBGYN= Physician specializing in obstetrics and gynecology, PSQI = Pittsburgh Sleep Quality Index

Supplemental Table 2: Abstracted data from studies included in meta-analysis

**Author, year, location, collection period,**	**Study Design, Sample, Exclusion criteria,**	**Exposure measurement:**<br><br>**Diagnosis criteria Outcome Scale**	**Results**	**Covariate other exposures or outcomes**
Álvarez-Salvago, 2020 [42]<br>Spain<br>Jan 2018-Jan 2019	Case Control<br>25 case 25 ctl<br>25-50 years of age	Clinical diagnosis, experiencing symptoms, >3 mo. since last surgery<br><br>Controls matched on age and height, evaluated by prof	Case 8.32 ± 3.72<br><br>Control 6.00 ± 3.19<br><br>P 0.017	Fatigue (sig)<br><br>Health Related Fitness (sig)<br><br>QoL (sig)

		and ultrasound to establish non-endo<br><br>Pittsburgh Sleep Quality Index-Spanish		
Chaichian, 2024 [43]<br>Tehran, Iran<br>2019–2022	analytic cross-sectional study<br><br>463 cases<br>202 ctl<br><br>18-40 y/o<br>(mean 35.4 SD 7.9)<br><br>Exclusion criteria were patients with previously known mental, neurologic, or sleep disorders, women with night shift work, use of medications with impact on the sleep-wake cycle.<br><br>Power calculated at min 400 for the case group	diagnosed with endometriosis by clinical signs and symptoms and ultrasonic findings<br><br>Pittsburgh Sleep Quality Index (PSQI) -<br>Persian version	Mean global PSQI cases: 10.6<br><br>Controls: 7.1<br>p<0.001<br><br>Effect size: 0.97<br>(0.79,1.14)	marital status, age, job, gravid, parity, BMI, infertility as independent variables in both groups and marital status, age, job, work, gravid, parity, BMI, infertility, dysmenorrhea, dyspareunia, dysuria, Pelvic pain, dyschezia, underlying diseases, hormonal, and surgical treatment as independent variables.
Facchin, 2021 [44]<br>Italy<br>July 2019 - March 2020	Matched pair case-control<br><br>123 case 123 controls (matched age and BMI)<br><br>21-45 y/o (34 SD 6.37)<br><br>current pregnancy; children <2 yo night shifts; and diagnosed cardiovascular, respiratory, renal, hepatic, gastrointestinal and/or psychiatric conditions	Cases were women with a surgical diagnosis of endometriosis in the previous 24 months or with a current clinical diagnosis of endometriosis.<br><br>Controls were women attending the study outpatient clinic for periodic gynecological care, cervical cancer screening programs<br>and contraception.<br><br>Pittsburgh Sleep Quality Index	Case<br><br>6.68<br>3.59<br><br>Control<br><br>5.45<br>3.03<br><br>P 0.004	age, employment status [employed/unemployed], presence of children [yes/no], BMI, smoking habits), clinical information (form of endometriosis, hormonal treatment, use of painkillers

Iannuzzo, 2024 [45]<br>Italy<br>Jan 2021-June 2021	Case Control<br>430 case 417ctl<br>presence, at the time of the interview, of psychiatric disorders on drug treatment, history of alcohol or substance addiction, and organic brain disorder or mental retardation.	Patient directory of endo patients, 357 had surgery 73 were based on symptoms<br><br>Controls from general population	Reported median IQR<br>Case 11 (7-16)<br><br>Control 5(3-8)<br><br>Mean SD after calculation<br>Case 11 (6.67)<br>Control 5 (2.20)	age, weight, height, fertility state, parity, and history of assisted reproductive techniques.<br><br>Hyperarousal scale, daytime sleepiness, insomnia
Nunes, 2015 [46]<br>Brazil<br>(no years reported)	case-control<br><br>257 cases 253 control<br><br>Mean age cases 34.4 SD 5.7<br>Controls 33.3 SD 6.2<br><br>Pregnancy, recent physical trauma and skin lesions on any of the body areas to be examined during data collection. Other criteria were the use of analgesics, anti-inflammatories and/or muscle relaxants in the 24 h preceding the study evaluation; use of corticosteroids in the previous week; use of psychotropic drugs, GnRH analogues and/or hormone therapy in the previous 3 months; history of hysterectomy or oophorectomy; or illicit drug use. Additionally, women reporting pain associated with active myofascial trigger points during physical examination, i.e., pain that moved or spread to a body region adjacent to that being examined, were excluded.<br><br>The sample size was calculated at 253 women in each group	laparoscopic and histopathological diagnosis of endometriosis<br><br>Post-Sleep Inventory (PSI)<br><br>Higher scores better quality sleep	Significant reduction in sleep quality case (5.68 ± 1.55) compared to control (6.04 ± 1.62) ($p = 0.011$).	demographic characteristics of the participants such as age, body mass index (BMI; kg/m2) and number of children were evaluated using Student's t-test, Mann–Whitney test and chi-square test, as appropriate

	for an α error of 0.05 and a β error of 0.20			
Youseflu, 2020 [47]<br>Iran<br>May 2016- Feb 2017	Case Control<br>78 case 78 ctrl<br><br>Excluded those with non-endo abnormalities found in lap from both cases and controls, history of chronic diseases or mental disorder according to self-report, occurrence of bad events in the 12 months ago (e.g. death or illness of family members or close friends, financial difficulties, ect)	Laparoscopic diagnosis<br><br>matched on age, education, and duration of infertility<br><br>Pittsburgh Sleep Quality Index-Persian	Case 6.47 ± 3.34<br><br>Ctrl 4.45 ± 3.26<br><br>P < 0.001	Dietary, socio demo, and clinical within patients were also examined<br><br>BMI examined found to be non-significant

Ctl = control, Mo= month, Endo= Endometriosis, SD = Standard Deviation, BMI= Body Mass Index, HT= Hormonal Therapy, IRQ = Interquartile Range, QoL= Quality of Life Y/O= years old, PSQI = Pittsburgh Sleep Quality Index, Sig= statistically significant
